# Undifferentiated spondyloarthritis following allogeneic stem cell transplantation

**DOI:** 10.1186/1471-2474-11-132

**Published:** 2010-06-25

**Authors:** Vijay R Karia, Raquel Cuchacovich, Luis R Espinoza

**Affiliations:** 1 Section of Rheumatology, Department of Internal Medicine, Louisiana State University Health Sciences Center, 1542 Tulane Avenue, New Orleans, LA 70112-2822, USA

## Abstract

**Background:**

Stem cell transplant has been utilized in the treatment of malignancies and rheumatic disease. Rheumatic disease may be transferred from the donor with active disease or may be developed in a recipient de novo as a late complication of SCT.

**Case Presentation:**

We here report the rare case of a 26-year old male patient, who has been diagnosed with undifferentiated spondyloarthropathy after unique circumstance. The patient suffered from intermittent inflammatory back pain and peripheral joint swelling for several years and did not find relief through multiple emergency room visits at different medical facilities. After a thorough history and physical exam, it was noted that our patient had developed signs of axial disease along with dactylitis and overall that he had been insidiously developing an undifferentiated spondyloarthopathy after allogeneic stem cell transplantation.

**Conclusion:**

Our observation supports the hypothesis that de novo rheumatic disease can develop after stem cell transplant for a variety of reasons. Thus, larger studies and awareness of this association are needed to delineate the exact underlying mechanism(s).

## Background

Both autologous and allogeneic stem cell transplantation (SCT) have become standard therapeutic modalities for lymphoid proliferative and certain autoimmune/rheumatic disorders [1,2]. With modified protocols to ensure less profound depletion of T cells, better control of systemic disease prior to transplantation, antiviral prophylaxis after transplantation, and slower tapering of corticosteroids both morbidity and mortality complications following SCT have significantly declined in recent years [3]. The development, however, of de novo rheumatic disorders is being increasingly recognized following either autologous or allogeneic SCT procedures [4-6]. The purpose of this report is to describe a patient in whom undifferentiated spondyloarthritis developed following allogeneic SCT.

Some rheumatic diseases appear to be directly related to SCT itself, while others may be associated to the therapy used (Additional File 1). Wagener et al. first brought into attention the development of musculoskeletal complaints following allogeneic but not autologous bone marrow transplantation [4]. Oligoarthritis occurring in 18/33 and avascular bone necrosis in 8/33 patients were noted. Since then several case reports or small series have appeared describing the development of a variety of rheumatic disorders including rheumatoid arthritis, HLA-B27 related spondyloarthritis, psoriatic arthritis, vasculitides syndromes including ANCA-associated small vessel vasculitis, eosinophilic fasciitis, ANA positivity, and antiphospholipid syndrome [7-12]. Gouty and acute inflammatory arthritis following the use of recombinant granulocyte colony-stimulating factor therapy has also been described in SCT patients [13,14]. Immune-mediated thyroid disease and cytopenias including Evans syndrome, hemolytic anemia, and thrombotic thrombocytopenic purpura (TTP)-like syndromes constitute another group of disorders seen in SCT [12,15].

A literature search along with consultation of leading hematologists was performed to find the current level of science in stem cell transplantation. Through our work, we found that a few other authors have noticed the phenomenon of autoimmune disease transmission after stem cell transplantation.

Our patient exhibits clinical manifestations associated with undifferentiated spondyloarthropathy [16]. HLA-B27 was not detected, but the presence of oligoarthritis, unilateral sacroiliitis, dactylitis, and enthesitis and a positive family history of psoriasis fulfill classification criteria for spondyloarthritis-most likely undifferentiated spondyloarthritis.

## Case Presentation

**Figure 1:**
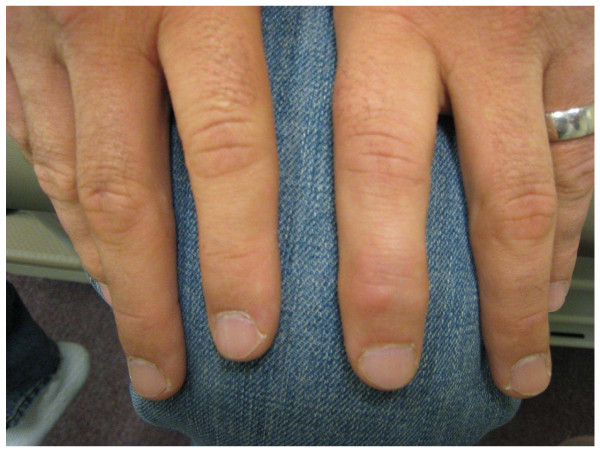
**Left index finger demonstrating DIP involvement**.

**Figure 2:**
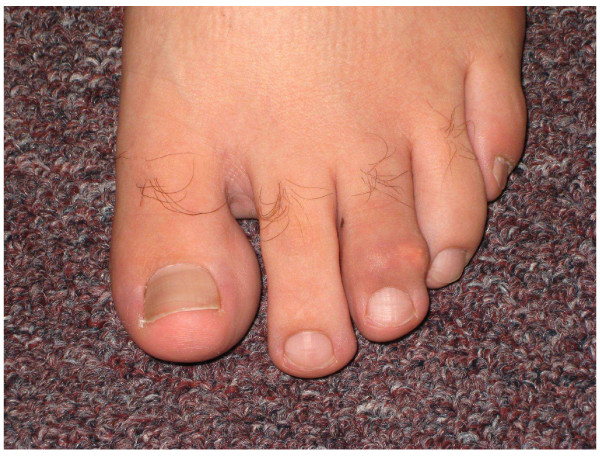
**Left foot exhibiting diffuse swelling with prominent DIP involvement**.

**Figure 3:**
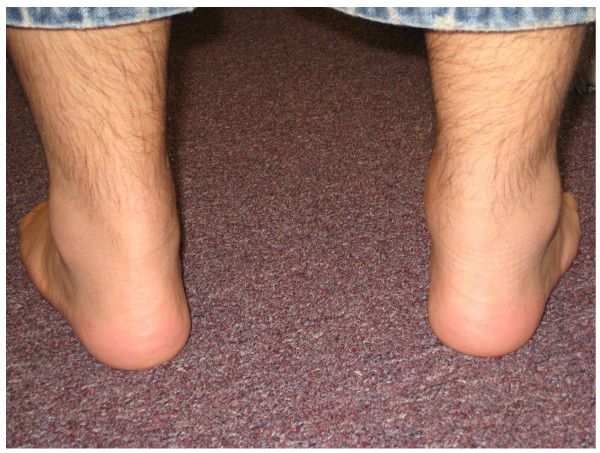
**Posterior view of ankles showing swelling of the right Achilles tendon and periarticular swelling**.

**Figure 4:**
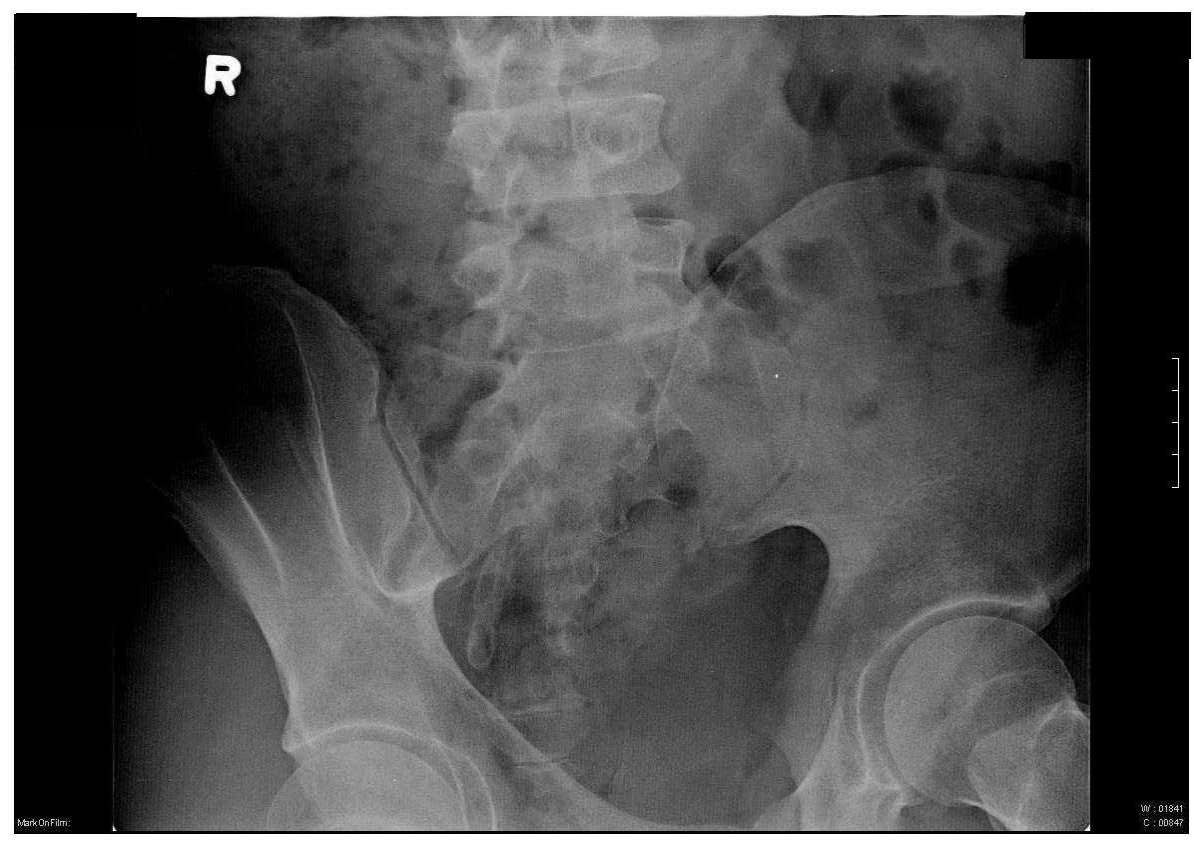
**Sacroiliac joints x-ray showing narrowing and irregularity of the left sacroiliac joint**.

A 26-year-old white man diagnosed with acute pre-B lymphocytic leukemia (ALL) at age 10 refractory to chemotherapy for which he underwent a haplo-identical allogeneic stem cell transplantation (SCT) from his father who did not have any rheumatic and/or dermatologic inflammatory diseases including psoriasis. Except for a febrile and convulsive episode that lasted only a few days with a very good response to a short course of oral prednisone, he remained well until about 10 years later when he began to exhibit lower back pain and stiffness. On Christmas day 2003, he woke up with a painful, stiff and swollen right ankle which made walking very difficult. From December 2003 onwards the patient complained of right ankle pain, swelling and stiffness with difficulty in walking and only partial and transient clinical response to indomethacin, colchicine and allopurinol therapy. In January 2008, he presented to a local emergency room with a painful, stiff, and swollen right elbow for which he received a short course of oral prednisone. Over the subsequent 4 months, he continued to have right elbow and ankle pain and swelling accompanied by difficulty using his arm and walking. In May 2008, he was admitted to our University Hospital for left elbow and hand swelling and pain. On physical examination, blood pressure was 131/73, pulse 81, height 5'8", weight 215, BMI 31.6. He was in distress and pertinent findings revealed the presence of mild flaking and dryness of earlobes overlying skin, swelling, redness and tenderness of the second digit distal interphalangeal joint of left hand, swelling, tenderness, and decreased range of motion of right ankle, sausage digit deformity of the middle digit of the left foot, and enthesitis of the right Achilles tendon (Figures 1, 2, 3). In addition, both sacroiliac joints were tender to palpation and a Schober test was positive. Past history: Hypertension, bilateral cataracts, and metabolic syndrome. Family history: Paternal grandfather had psoriasis and also suffered from stiff hands. Maternal grandmother had a history of rheumatoid arthritis for over 30 years. Laboratory findings: ESR: 56 mm/h (n < 20), CRP: 17.02 mg/dL (n < 0.80). Rheumatoid factor, CCP antibodies, ANA, and HLA-B27 were negative and/or normal. Hands and feet radiographs did not show erosions. Sacroiliac joints x-ray showed unilateral sacroiliitis (Figure 4). Follow-up: patient is improved on symptomatic NSAID therapy.

## Conclusion

We present an interesting patient who has undergone stem cell transplantation for the treatment of pre-B lymphocytic leukemia and was in remission. However, his development of a de novo rheumatic disease was recognized by us and the incidence is increasing according to recent medical literature. We reviewed current theories regarding the reasons behind autoimmune disease transfer between donor and recipient and postulate that our patient fits into the current science.

One could consider the development of de novo autoimmune disease as a possible side effect of stem cell transplantation. These serious secondary effects of stem cell transplantation must be taken into account as tertiary centers continue using SCT as a modality for the treatment of rheumatic and hematologic disease.

The exact pathophysiology of both rheumatic and immune-mediated disorders is not fully understood. It is quite likely, however, that multiple factors including genetic predisposition, environmental factors such as CMV infection, drug exposure, abnormal reconstitution of the immune system, and direct transfer of pathogenic hematopoietic stem cells from the donor that could generate autoreactive clones in allogeneic transplants-possibly the case in our patient, might participate. Inflammatory cytokines, chemokines and adhesion molecules, particularly interleukin-8 and drugs used during SCT such as cyclosporine A or tacrolimus may also play an important role in inducing vascular endothelial damage leading to TTP-like syndromes.

Our patient revealed a clinical picture consistent with undifferentiated spondyloarthritis which developed following allogeneic SCT. The exact incidence as well as the pathophysiology of this complication is unknown, but larger studies are needed to develop preventive measures.

## Abbreviations

SCT: stem cell transplant.

## Competing interests

The authors declare that they have no competing interests.

## Authors' contributions

VRK was an active physician in this patient's management and completed the background research and initial drafts for this manuscript. RC was an active physician in this patient's management and performed background literature search. LRE was an active physician in this patient's management, conceived of the study, and participated in its design and coordination. All authors read and approved the final manuscript.

## Consent

Patient has given authors consent for the use of these clinical images for publication.

## Pre-publication history

The pre-publication history for this paper can be accessed here:

http://www.biomedcentral.com/1471-2474/11/132/prepub

## Supplementary Material

Additional file 1 **Supplemental table**. Rheumatic and immune-mediated disorders following both autologous and allogenic stem cell transplantation. Click here for file
