# Supplementary material for: Undifferentiated spondyloarthritis following allogeneic stem cell transplantation
Source: BMC Musculoskelet Disord. 2010 Jun 25;11:132. doi: 10.1186/1471-2474-11-132 (PMC2902413; doi:10.1186/1471-2474-11-132)
Supplement: Additional file 1 — Supplemental table. Rheumatic and immune-mediated disorders following both autologous and allogenic stem cell transplantation. [file 1471-2474-11-132-S1.DOC]

**TABLE 1**.

RHEUMATIC AND IMMUNE-MEDIATED DISORDERS FOLLOWING BOTH AUTOLOGOUS AND ALLOGENIC STEM CELL TRANSPLANTATION

Rheumatic Disorders:

- Rheumatoid arthritis
- HLA-B27-associated spondyloarthropathy
- Psoriasis and psoriatic arthritis
- Undifferentiated spondyloarthropathy
- Oligoarthritis
- Eosinophilic fasciitis
- Vasculitis: ANCA-associated
- Anti-phospholipid antibody syndrome
- Avascular bone necrosis
- Drug-induced: G-CSF-induced gout and acute arthritis

Immunune-mediated Disorders:

- Autoimmune thyroid dysfunction
- Autoimmune cytopenias: Thrombotic thrombocytopenic-like purpura,

Coombs positive hemolytic anemia, lymphopenia, Evans syndrome, thrombocytopenia
